# Population structure and plumage polymorphism: The intraspecific evolutionary relationships of a polymorphic raptor, *Buteo jamaicensis harlani*

**DOI:** 10.1186/1471-2148-10-224

**Published:** 2010-07-22

**Authors:** Joshua M Hull, David P Mindell, Sandra L Talbot, Emily H Kay, Hopi E Hoekstra, Holly B Ernest

**Affiliations:** 1 Wildlife and Ecology Unit, Veterinary Genetics Laboratory, University of California, One Shields Avenue, Davis, CA 95616, USA; 2 Genomic Variation Laboratory, Department of Animal Science, University of California, Davis, CA 95616, USA; 3 California Academy of Sciences, 55 Music Concourse Drive, San Francisco, CA 94118, USA; 4 Alaska Science Center, US Geological Survey, 4210 University Drive, Anchorage, AK 99508, USA; 5 Department of Organismic and Evolutionary Biology, Museum of Comparative Zoology, Harvard University, 26 Oxford Street, Cambridge, MA 02138, USA; 6 Department of Population Health and Reproduction, School of Veterinary Medicine, University of California, One Shields Avenue, Davis, CA 95616, USA

## Abstract

**Background:**

Phenotypic and molecular genetic data often provide conflicting patterns of intraspecific relationships confounding phylogenetic inference, particularly among birds where a variety of environmental factors may influence plumage characters. Among diurnal raptors, the taxonomic relationship of *Buteo jamaicensis harlani *to other *B. jamaicensis *subspecies has been long debated because of the polytypic nature of the plumage characteristics used in subspecies or species designations.

**Results:**

To address the evolutionary relationships within this group, we used data from 17 nuclear microsatellite loci, 430 base pairs of the mitochondrial control region, and 829 base pairs of the melanocortin 1 receptor (*Mc1r*) to investigate molecular genetic differentiation among three *B. jamaicensis *subspecies (*B. j. borealis*, *B. j. calurus*, *B. j. harlani*). Bayesian clustering analyses of nuclear microsatellite loci showed no significant differences between *B. j. harlani *and *B. j. borealis*. Differences observed between *B. j. harlani *and *B. j. borealis *in mitochondrial and microsatellite data were equivalent to those found between morphologically similar subspecies, *B. j. borealis *and *B. j. calurus*, and estimates of migration rates among all three subspecies were high. No consistent differences were observed in *Mc1r *data between *B. j. harlani *and other *B. jamaicensis *subspecies or between light and dark color morphs within *B. j. calurus*, suggesting that *Mc1r *does not play a significant role in *B. jamaicensis *melanism.

**Conclusions:**

These data suggest recent interbreeding and gene flow between *B. j. harlani *and the other *B. jamaicensis *subspecies examined, providing no support for the historical designation of *B. j. harlani *as a distinct species.

## Background

The criteria necessary to recognize and define distinct species have been frequently debated [1-3]. One of the primary difficulties in species designation is that the process of speciation requires an extended period of time during which various attributes of species-level distinction are attained [2]. Depending on the length of time since divergence, only some fraction of phenotypic and molecular characters may have become fixed between incipient species. Therefore, different characters and associated species concepts may provide conflicting inference in the determination of species status [e.g.,[4,5]]. For example, the relative influence of genetic drift versus natural selection, relative sizes of populations sampled, and age of barriers to reproduction (if any) of the species involved will affect the degree to which characters become diagnostic, thus influencing phylogenetic inference. One manifestation of this phenomenon occurs during the comparison of phenotypic and molecular genetic data where conflicting patterns of divergence are detected [see [4,6]]. Attempts to accurately define evolutionary relationships are further confounded if expression of the phenotypic trait examined is influenced by environmental factors apart from any genomic control and therefore does not faithfully reflect evolutionary relationships.

Understanding the relationship between phenotypic and molecular genetic data sets may be particularly informative in studies of avian taxonomy where plumage characteristics have been historically used to designate subspecies as well as species [7]. Many species and subspecies designations based upon phenotype alone have not been supported by molecular genetic phylogenies [e.g.,[8,9]]. In fact, among avian researchers, the value of designating subspecies rank is subject to ongoing debate often as vigorous as the debate over species concepts [6,10]. Inconsistencies at both hierarchical levels are associated with the difficulty in understanding the underlying genetic and/or environmental basis of phenotypic characters, and consequently, interpreting phenotypic patterns alone may mislead evolutionary inference [6,10]. Phenotypic characters associated with melanin-based plumage may be particularly susceptible to misinterpretation in an evolutionary context due to environmental and temporal influences. Expression of melanin-based characters can vary with body condition [11,12], oxidative stress induced by environmental factors [13], and temporally within individuals [14].

Among diurnal raptors, phenotypic differences have fueled a long-standing controversy concerning intraspecific relationships (i.e., subspecies designations) within the North American raptor *Buteo jamaicensis *(red-tailed hawks). This controversy has been motivated primarily by the presence of substantial variation in plumage characteristics of *B. j. harlani*, of which almost all individuals display melanistic plumage. By contrast, the majority of *B. j. calurus *are not melanistic, and melanistic plumage has not been documented in either *B. j. alascensis *or *B. j. borealis *[15,16]. Other melanin-based plumage characters, such as tail pattern and coloration and throat coloration, show extreme variation within *B. j. harlani *but overlap to some degree with plumage characters observed in other *B. jamaicensis *subspecies [17]. The phenotypic variation within *B. j*. *harlani *has been cited as evidence to consider it as either a distinct species, *B. harlani *[18], or as a subspecies within *B. jamaicensis *[19-21]. Whether observed plumage differences within *B. jamaicensis *reflect long-standing evolutionarily distinct lineages or are a consequence of environmental, temporal, or physiological factors, particularly within the range of *B. j. harlani*, remains undetermined.

Previous research has examined the phylogeographic relationships of *B. j. calurus *and *B. j. borealis *across nine breeding populations and ten migration sites [22]. This earlier analysis of microsatellite data in *B. jamaicensis *indicated low levels of genetic differentiation among breeding sites within recognized subspecies. Fine-scale population substructure was identified between central California and Intermountain West populations of *B. j. calurus *(F_ST _= 0.011 - 0.018) [22]; no other significant within-subspecies differentiation was identified. Significant differentiation was also identified between *B. j. borealis *and *B. j. calurus*, supporting historical subspecies designations (F_ST _= 0.036 - 0.039) [22,23].

Building upon this previous work, we used molecular genetic data from the mitochondrial and nuclear genomes to investigate the taxonomic relationship of *B. j. harlani *to two common and widespread subspecies geographically adjacent to *B. j. harlani*; *B. j. borealis *and *B. j. calurus*. Specifically, we investigated whether *B. j. harlani *is distinct at the species level by comparing our results with the levels of differentiation previously documented among subspecies of *B. jamaicensis*. If *B. j. harlani *represents a distinct species, it should at a minimum conform to criteria proposed for assessing status as a subspecies, or an "evolutionary significant unit," [24-26]. These include reciprocal monophyly at mitochondrial DNA (mtDNA) and significant partitions at nuclear loci, relative to the other named subspecies. The results of our analyses help to resolve a long-standing taxonomic challenge and provide insight into the role of plumage variation in species designations.

## Methods

### Sample collection

We collected whole blood and feathers during the breeding season (May through June) from young in nests and adults on breeding territories of three putative *B. jamaicensis *subspecies; 178 *B. j. calurus*, 69 *B. j. borealis*, and 24 *B. j. harlani *(Table 1). Additionally, we collected samples from 21 melanistic individuals, presumably *B. j. calurus*, during autumn migration. We drew approximately 0.2 mL of blood from the medial metatarsal vein and plucked two feathers from the breast. We received approval for our methodology from the U.C. Davis Institutional Animal Care and Use Committee (protocol number 12260). We stored blood samples in 1.5 mL of Longmire's lysis buffer (100 mM Tris pH 8.0, 100 mM EDTA, 10 mM NaCl, 0.5% SDS) at ambient temperature in the field and at -80°C once delivered to the laboratory and we stored feathers in envelopes in cool and dry conditions. We extracted genomic DNA from 25 μL of blood/buffer solution or a single feather calamus using Qiagen DNeasy kits (Qiagen Inc.); we stored DNA at -80°C following extraction.

**Table 1:** Sampling locations and the number of individuals for *B. j. calurus*, *B. j. borealis*, *B. j. harlani*, and *B. jamaicensis *spp (melanistic).

Subspecies	Location	N
*B. j. calurus*	central California	68
	Idaho	35
	Nevada/Utah	39
	southern California	36
		
*B. j. borealis*	Alberta	2
	Colorado	11
	Kentucky	9
	Michigan	5
	north Texas	9
	Wisconsin	33
		
*B. j. harlani*	Anchorage, Alaska	6
	Fairbanks, Alaska	3
	Kuskokwim, Alaska	10
	Palmer, Alaska	4
	Kluane, Yukon Territory	1
		
*B. jamaicensis *spp. (melanistic)	central California	21

### Microsatellite data collection and analysis

We genotyped each individual at 17 microsatellite loci (BswA110w, BswA204w, BswA302w, BswA303w, BswA312w, BswA317w, BswB111aw, BswB220w, BswB221w, BswD122w, BswD127w, BswD210w, BswD220w, BswD234w, BswD310w, BswD313w, BswD327w) in six multiplex reactions following previously developed methods [27]. We separated PCR products using a 3730 DNA Analyzer (Applied Biosystems Inc.) and scored products using STRAND version 2.3.89 [28].

Because of the limited genetic differentiation previously documented among breeding populations within *B. j. calurus *and *B. j. borealis *[23] and the focus of our study on the taxonomic status of *B. j. harlani *in relation to other subspecies, we treated subspecies as our unit of analysis for the majority of the population genetic analyses. We tested for deviations from Hardy-Weinberg equilibrium in all loci using GENEPOP version 3.4 [29] and we tested each pair of loci in each putative subspecies (*B. j. calurus*, *B. j. borealis*, and *B. j. harlani*) for genotypic disequilibrium in GENEPOP. After we assessed significance of pairwise tests using a sequential Bonferroni correction for multiple comparisons [30], we tested for the presence of null alleles and scoring error in MICROCHECKER [31]. Previous research has indicated that the rate of scoring error for this set of microsatellites is 0.8% [23].

We used the program CONVERT version 1.31 [32] to determine the number of private alleles in each presumed subspecies. We used MICROSATELLITE TOOLKIT [33] to calculate observed and expected heterozygosity of the total sample and within each subspecies as well as the average number of alleles per locus. Allelic richness, weighted by sample size, was estimated for each subspecies using FSTAT version 2.9.3.2 [34].

We determined pairwise estimates of variance in allele frequency (θ_ST_) for all subspecies pairs in ARLEQUIN version 3.11 [35] and we used a sequential Bonferroni correction for multiple tests to determine significance of θ_ST _values [30]. We analyzed the relationships between sampling localities with greater than 20 individuals by averaging allele frequency differences among sampling sites [36]. We used the resulting divergence matrices with both neighbor-joining and UPGMA algorithms to make a clustering tree in the program PHYLIP [37] and assessed node support using 10,000 bootstrap replicates.

In order to further test for a distinction between subspecies and investigate the possibility of cryptic population differentiation beyond subspecies groupings, we implemented a Bayesian clustering analysis in STRUCTURE version 2.1 [38]. The unit of analysis was the individual and the algorithm implemented in Structure probabilistically grouped individuals together into the most likely number of populations without regard to presumed subspecies. This approach allowed us to determine the most likely number of population groups (K) without *a priori *information about subspecies or region of origin. We used the population admixture model with a flat prior and assumed allele frequencies were correlated. We allowed the program to run with a 500,000 iteration burn-in and a run length of 1,000,000 iterations and used this parameter set to explore K = 1 through K = 5 and averaged log Pr(X|K) estimates across 10 runs for each value of K. We determined the most likely number of populations by selecting the set of K values where the log Pr(X|K) estimation was maximized and then selecting the minimum value for K that did not sacrifice explanatory power [39,40]. We grouped individuals into clusters based upon the highest proportion of ancestry to each inferred cluster.

### Mitochondrial data collection

We sequenced a 430 base pair segment of domain I of the mitochondrial control region using primers 16065F [41] and H15454 [42]. We prepared PCR products for sequencing with 0.5 μL exonuclease I and 1 μL shrimp alkaline phosphatase per 25 μL PCR and delivered PCR products to the UC Davis Sequencing Facility using primers 14965F and H15414 [43]. We aligned sequences by eye using SEQUENCHER version 4.7 (Gene Codes Corporation).

### Phylogeographic analyses of mtDNA sequence

We conducted phylogenetic analyses of control region sequences in PAUP* version 4.0b8 [44], using maximum parsimony (MP), maximum likelihood (ML) and distance (minimum evolution, ME). We used MODELTEST version 3.06 [45] to determine the minimum parameter nucleotide substitution model that best fit the mtDNA sequence data under the Akaike Information Criterion [46]. In each analysis, we conducted heuristic tree searches, with 20 and 100 random additions of taxa for maximum likelihood and parsimony analyses, respectively, each followed by tree bisection-reconnection topological rearrangements. We applied midpoint rooting, employing the close-neighbor interchange algorithm for finding the tree, beginning with an initial neighbor-joining tree. We assessed the robustness of nodes using tree reconstructions of bootstrap-resampled data sets for 1,000 replicates under distance and parsimony criteria, and 200 replicates for ML criteria.

We calculated the number of variable sites, number of haplotypes, haplotype diversity, and nucleotide diversity in DNAsp [47]. Due to the low levels of divergence within subspecies previously documented [22], we calculated pairwise estimates of variance in haplotype frequency (Φ_ST_) between each presumed subspecies using haplotype frequencies in ARLEQUIN version 3.11 and we assessed significance using 10,000 permutations. We used a sequential Bonferroni correction for multiple tests to determine significance of Φ_ST _values [30].

### Estimation of polarity in gene flow

To provide a coarse estimate of gene exchange among subspecies, we calculated the number of migrants per generation (*N*_*e*_*m*) for nuclear microsatellite and number of female migrants per generation (*N*_*f*_*m*) for mtDNA among populations using MIGRATE version 2.0.6 [48-50]. To test for asymmetry in gene flow, we estimated full models, θ (4*N*_*e*_μ or *N*_*f*_μ, composite measure of effective population size and mutation rate), and all pairwise migration parameters individually from the data and compared them to a restricted island model for which θ was averaged and pairwise migration parameters were symmetrical between populations.

We ran MIGRATE using maximum likelihood search parameters; ten short chains (1,000 used trees out of 20,000 sampled), five long chains (10,000 used trees out 200,000 sampled), and five adaptively heated chains (start temperatures: 1, 1.5, 3, 6, and 12; swapping interval = 1). We ran full models three times to ensure the convergence of parameter estimates; we ran restricted models once. We evaluated the alternative model for goodness-of-fit given the data using a log-likelihood ratio test. The resulting statistic from the log-likelihood ratio test is equivalent to a χ^2 ^distribution with the degrees of freedom equal to the difference in the number of parameters estimated in the two models [50].

### Melanocortin 1 receptor sequencing

To determine if differences in the coding region of *melanocortin 1 receptor *(*Mc1r*) gene are associated with subspecies or color variation in *B. jamaicensis*, we sequenced *Mc1r *from 23 individuals (17 *B. j. calurus; *6 *B. j. harlani*) using primers MSHR9 and MSHR72 [51]. We amplified an 829 bp fragment from the 945 bp avian *Mc1r *coding region, excluding 56 bases from the 5' end and 54 bases from the 3' end of exon 1. We used the following polymerase chain reaction (PCR) conditions for a 15 μL total reaction: 1.5 μl 10× reaction buffer, 0.3 μL of dNTPs (10 μM), 0.5 μL each primer (10 μM), 0.1 μL Taq polymerase, and 20 ng DNA. We used the following PCR cycling parameters: 94°C for 3:00, (94°C for 0:30, 61°C for 0:45, 72°C for 1:00) × 40 cycles, 72°C for 10:00, 15°C forever. We performed cycle sequencing for both strands with Big Dye v. 2 using the amplification primers and two additional internal primers (intF 5'-AGCAACCTGGCAGAGACACT-3' and intR 5'-TGTAGAGCACCAGCATGAGG-3'; 4 μM) following standard sequencing protocols. We sequenced all PCR products on an ABI PRISM^® ^3100 Genetic Analyzer (Applied Biosystems, Foster City, CA) and edited the sequences in the program Sequencher version 4.7 (Gene Codes Corporation). We identified all heterozygous sites by visual inspection of chromatogram peaks and confirmed each site using sequences from both strands.

## Results

### Microsatellite data

None of the 17 microsatellite loci significantly differed from Hardy-Weinberg equilibrium expectations and we detected no evidence of linkage disequilibrium. We found no evidence of null alleles. Observed heterozygosity, average number of alleles, private alleles, and allelic richness for each sampling site are summarized in Table 2.

**Table 2:** Genetic diversity indices for microsatellite and mitochondrial sequence data.

	N_μsat_	H_O _± sd	A_a _± sd	A_p_	A_r_	N_mt_	H_N_	H_P_	π	Hd
*B. j. calurus*	178	0.68 ± 0.01	14.2 ± 10.3	34	6.9	122	14	8	0.0019	0.57 ± 0.003
central California	68	0.68 ± .01	11.4 ± 7.6	9	6.3	29	6	2	0.0012	0.43 ± 0.11
Idaho	35	0.66 ± 0.02	10.1 ± 6.3	5	6.5	34	6	2	0.0012	0.37 ± 0.10
Nevada/Utah	39	0.71 ± 0.02	11.2 ± 7.2	9	6.8	29	8	1	0.0016	0.57 ± 0.11
southern California	36	0.65 ± 0.02	9.6 ± 5.1	3	6.5	30	4	0	0.0009	0.35 ± 0.10
										
*B. j. borealis*	69	0.73 ± 0.01	12.1 ± 7.9	11	7.1	52	13	8	0.0019	0.520 ± .007
Alberta	2	0.79 ± 0.07	2.9 ± 1.1	0	---	---	---	---	---	---
Colorado	11	0.73 ± 0.03	7.1 ± 3.4	1	6.6	6	1	0	---	---
Kentucky	9	0.72 ± 0.04	6.2 ± 2.5	0	---	10	5	1	0.0022	0.67 ± 0.16
Michigan	5	0.76 ± 0.05	5.1 ± 2.0	1	---	5	3	1	0.0023	0.70 ± 0.22
north Texas	9	0.73 ± 0.04	6.7 ± 3.4	1	---	5	2	1	0.0008	0.33 ± 0.22
Wisconsin	33	0.73 ± 0.02	9.8 ± 5.3	5	6.6	26	8	5	0.0022	0.47 ± 0.12
*B. j. harlani*	24	0.69 ± 0.03	9.0 ± 5.3	5	6.9	24	5	0	0.016	0.59 ± 0.05
*B. jamaicensis *spp.										
melanistic migrants	21	0.68 ± 0.03	9.0 ± 5.2	1	6.9	---	---	---	---	---

Key: N_μsat _= the number of samples for which microsatellite data was collected, H_O _= observed heterozygosity, A_a _= average number of alleles per locus, A_p _= number of private alleles, A_r _= allelic richness averaged across 17 loci, N_mt _= the number of samples for which sequence data was collected, H_N _= the number of haplotypes, H_P _= the number of private haplotypes, π = nucleotide diversity, Hd = haplotype diversity.

**Figure 1:**
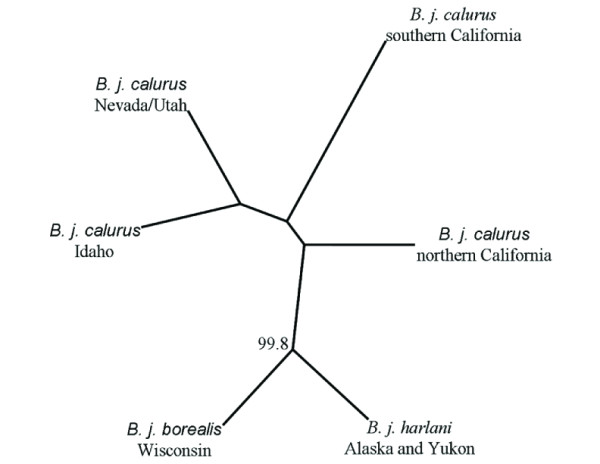
**Unrooted UPGMA clustering tree based on microsatellite allele frequency differences**. Only breeding sites with greater than 20 individuals were included; we assessed support using 10,000 bootstrapped data sets. Nodes with greater than 50% bootstrap support are indicated. The results shown here are consistent with previous research that found support in UPGMA clustering for a distinction between *B. j. calurus *and *B. j. borealis *breeding and migration sites [22].

Strong bootstrap support (99.8%) in the UPGMA tree (Figure 1) indicated a distinction between all *B. j. calurus *samples and the combined sample of *B. j. borealis *and *B. j. harlani*. No other nodes were supported by bootstrap analysis. Neighbor joining tree topology (not shown) was identical to that observed in the UPGMA tree. This result is consistent with previous UPGMA clustering that supported a distinction between *B. j. borealis *and *B. j. calurus *for all breeding and migration sites [22].

Pairwise θ_ST _values for 17 microsatellite loci among subspecies are summarized in Table 3. We observed significant differentiation between *B. j. calurus *and both *B. j. borealis *and *B. j. harlani*. However, we did not detect significant differences in variance in allele frequency between *B. j. borealis *and *B. j. harlani*. A sample of 21 melanistic individuals trapped in migration in central California, presumably *B. j. calurus*, was significantly different than both *B. j. harlani *and *B. j. borealis *individuals, but genetically indistinct from *B. j. calurus *individuals.

**Table 3:** Microsatellite pairwise θ_ST _comparisons between *B. jamaicensis *subspecies.

	*B. jamaicensis *spp. (melanistic)	*B. j. calurus*	*B. j. borealis*	*B. j. harlani*
*B. jamaicensis *spp. (melanistic)	----------			
*B. j. calurus*	0.004	----------		
*B. j. borealis*	0.023*	0.012*	----------	
*B. j. harlani*	0.023*	0.020*	0.004	----------

Key: *'s indicate significant differences following Bonferroni correction. Melanistic individuals were sampled during autumn migration within the range of *B. j. calurus*; however the sample may include individuals that originated outside the range of *B. j. calurus*.

Bayesian clustering analysis of breeding individuals without regard to subspecies or region of origin revealed a maximum log Pr(X|K) for K = 2 reflecting two distinct population clusters. One cluster corresponded to a group composed of *B. j. calurus *individuals while the second group was comprised of both *B. j. borealis *and *B. j. harlani*. As an additional test for the existence of a distinct *B. j. harlani *cluster we also examined population clustering for K = 3. We continued to find a distinct *B. j. borealis*/*harlani *cluster while the *B. j. calurus *cluster was further subdivided into a central California cluster and an intermountain desert cluster, consistent with the findings of Hull et al. 2008 [22]. Finally, we performed a separate run including only *B. j. borealis *and *B. j. harlani *samples to test whether any fine-scale structure could be identified. For this subset of the data, the maximum log Pr(X|K) occurred at K = 1, but was very similar to the value at K = 2. For K = 2, no pattern of cluster ancestry was apparent between *B. j. borealis *and *B. j. harlani*; a Yates corrected χ^2 ^test also showed no difference in the proportion of ancestry (χ^2^_1 _= 1.52, *P *= 0.22).

### Mitochondrial data

**Figure 2:**
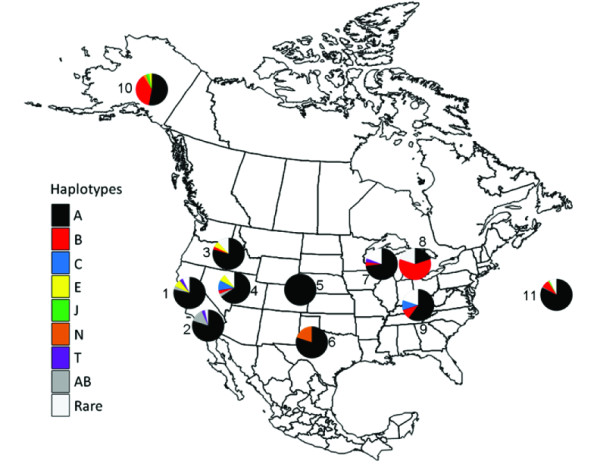
**Map of North American *B. jamaicensis *sampling locations**. We collected all samples from breeding sites during the breeding season from either young in nests or territorial adults. We collected samples from sites 1-4 within the breeding range of *B. j. calurus*; samples from sites 5-9 within the breeding range of *B. j. borealis*; samples from site 10 within the breeding range of *B. j. harlani*; and we collected the pooled samples from site 11 from migrants of unknown breeding origin, all samples from site 11 appeared phenotypically similar to typical or melanistic *B. j. calurus*. Sampling areas are shown with associated frequencies of mitochondrial control region haplotypes.

We collected control region sequence data for 198 individuals from presumptive populations of *B. j. borealis *(n = 52), *B. j. calurus *(n = 122), and *B. j. harlani *(n = 24) and observed 29 haplotypes (GenBank accession numbers HM454161 - HM454189). Of these haplotypes, eight were unique to *B. j. borealis *and five unique to *B. j. calurus*; we did not identify any unique haplotypes in *B. j. harlani*. Haplotype and nucleotide diversity were generally lower in *B. j. calurus *than in either *B. j. borealis *or *B. j. harlani *which were relatively similar to each other (Table 2). Distribution and frequency of control region haplotypes among sampling sites is shown in Figure 2. Pairwise Φ_ST _comparisons among subspecies revealed significant differentiation among all pairs of subspecies (Table 4).

**Table 4:** Mitochondrial pairwise Φ_ST _comparisons between *B. jamaicensis *subspecies

	*B. j. calurus*	*B. j. borealis*	*B. j. harlani*
*B. j. calurus*	----------		
*B. j. borealis*	0.032*	----------	
*B. j. harlani*	0.203*	0.059*	----------

Key: *'s indicates significant differences following Bonferroni correction.

Analysis of mtDNA sequence data suggested that the best model fit to the data under the hierarchical likelihood ratio criterion was the HKY+I_(0.90) _+G _(Γ = 0.85) _model [52]. Under the Akaike Information Criterion (AIC) criterion the best fit model was the K81uf+I_(0.90) _+G_(Γ = 0.85) _[53] model with an invariant site parameter (TrN+I). The log likelihood score for the HKY+I_(0.90) _+G _(Γ = 0.85 _model (-lnL = 769.17) was similar to that for the K81uf+I_(0.90) _+G_(Γ = 0.85) _model (-ln L 768.02, AIC = 1550.05). For the HKY+I model, we estimated the transition to transversion ratio at 14.42.

**Figure 3:**
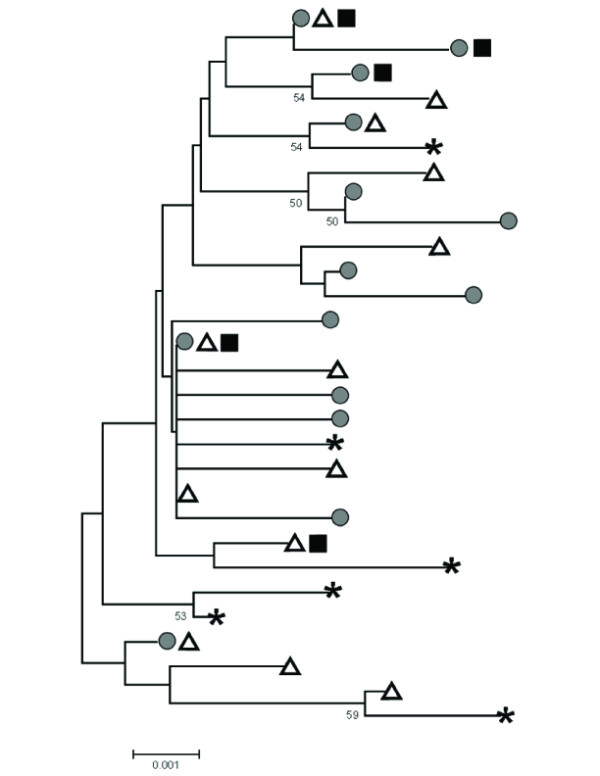
**Neighbor joining distance mid-point rooted minimum evolution tree of *B. jamaicensis *haplotypes**. Branch length indicates degree of genetic differentiation. Bootstrap values providing > 50% support for nodes are indicated. Gray circles indicate haplotypes sampled from breeding sites of *B. j. calurus*, white triangles indicate haplotypes sampled from breeding sites of *B. j. borealis*, black squares indicate haplotypes sampled from breeding sites of *B. j. harlani*, and asterisks indicate haplotypes that were sampled from migrant hawks in California with plumage consistent with *B. j. calurus*. We applied midpoint rooting, employing the close-neighbor interchange algorithm for finding the tree, beginning with an initial neighbor-joining tree.

All gene tree reconstruction methods showed results similar to the among-haplotype relationships shown in the ME tree (Figure 3): haplotypes did not form monophyletic clades with respect to subspecies, and node support was weak. Both ME and ML analyses gave marginal bootstrap support in few cases with no support greater than 59% (data for ME analysis only given in Figure 3).

**Figure 4:**
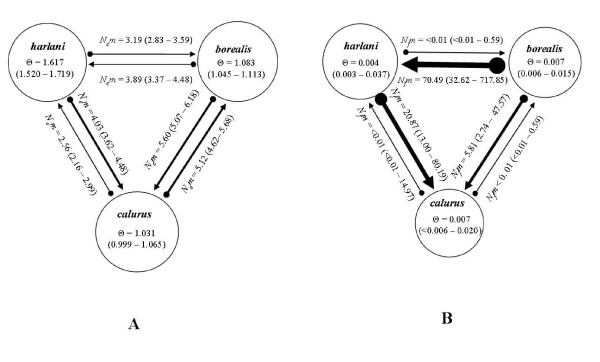
**Results of full model migration matrix**. We allowed all parameters to vary independently. We calculated migration direction and rates from: (A) 17 microsatellite loci and (B) mtDNA control region data. *N*_*e*_*m *and *N*_*f*_*m *= estimate for number of migrants/females per generation based on nuclear microsatellites and mtDNA, respectively. Θ = population size (4N_e_μ; see text). 95% confidence intervals for parameter values are in parentheses. Arrow thickness is proportionate to estimated levels of gene flow (thicker arrows indicate higher relative gene flow).

We observed asymmetrical gene flow among all subspecies and across both marker types. The full model (all parameters allowed to vary independently) had significantly higher likelihoods than the restricted island model (equal inter-group migration rate and equal θ across populations; microsatellite: LnL_restricted _= -1078.32, LnL_full _= -801.21, *P *< 0.0001; mtDNA: LnL_restricted _= -527.48, LnL_full _= 6.20, *P *< 0.0001). *N*_*e*_*m *and θ (4*N*_*e*_μ) values calculated in MIGRATE from microsatellite genotypes and mtDNA ranged from 2.56 -5.60 migrants per generation among subspecies, with θ ranging from 1.031 - 1.62 (Figure 4A), and <0.01 to 70.49 female migrants per generation among subspecies, with θ ranging from 0.004 - 0.007 (Figure 4B). The bias in the variances and the means indicate that, on average over generations, gene flow has been greater from *B. j. borealis *and *B. j. harlani *to *B. j. calurus *than *B. j. calurus *to the other two subspecies (Figure 4A, B). While gene flow estimates between *B. j. harlani *and *B. j. borealis *appear more symmetrical (overlapping confidence intervals) when based on microsatellite loci (Figure 4A), estimates based on mtDNA suggest significantly greater levels of gene flow from *B. j. borealis *to *B. j. harlani *than vice versa (Figure 4B). These estimates provide a rough estimation of the rate of gene exchange among subspecies and should not be viewed as precise measures. Rather, these results should be considered in the context of whether *B. j. harlani *is sufficiently isolated from other *B. jamaicensis *subspecies to be considered a full species.

### Mc1r results

We sequenced 829 base pairs corresponding to 276 out of 314 amino acids in the avian *Mc1r*, excluding 19 amino acids in the N-terminus domain and 17 amino acids in the C-terminus domain (GenBank accession numbers HM454190 - HM454192). We found that six internal nucleotides were absent relative to the *Gallus gallus Mc1r*. We found three synonymous substitutions within conserved transmembrane regions I and III, but no non-synonymous substitutions in the 829 bp of *Mc1r *sequenced from the 23 *B. jamaicensis *individuals sampled (Table 5). None of the three synonymous substitutions were consistently associated with rufous, dark, light, partial albino, or *B. j. harlani *color morphs. These data suggest that the *Mc1r *locus in *B. jamaicensis *is not responsible for breast color variation or the variable melanin-based plumage patterns observed in *B. j. harlani*.

**Table 5:** *Mc1r *polymorphism for breast color morphs in *B. j. calurus *and *B. j. harlani *subspecies.

Phenotype	Subspecies	Specimen no.	73	292	346
		**Consensus**	**G**	**C**	**C**

Rufous	*calurus*	10094	.	.	.
Rufous	*calurus*	13424	.	.	.
Rufous	*calurus*	13642	.	.	.
Rufous	*calurus*	13651	.	.	.
Rufous	*calurus*	13662	.	.	.
Rufous	*calurus*	13737	.	.	.
Rufous	*calurus*	13775	.	.	.
Rufous	*calurus*	13826	.	.	.
Dark	*calurus*	11071	.	.	.
Light	*calurus*	10101	G/A	.	.
Light	*calurus*	11056	.	.	.
Light	*calurus*	13670	.	.	.
Light	*calurus*	13671	.	.	.
Light	*calurus*	13672	.	C/T	.
Light	*calurus*	13673	.	.	C/T
Light	*calurus*	13674	G/A	.	.
Partial albino	*calurus*	6161	.	.	.

Harlan's	*harlani*	10983	.	.	.
Harlan's	*harlani*	13654	.	C/T	.
Harlan's	*harlani*	14133	.	.	C/T
Harlan's	*harlani*	14134	.	.	.
Harlan's	*harlani*	14138	.	.	.
Harlan's	*harlani*	14139	.	.	.

		**AA**	**Thr**	**Ile**	**Ile**
		**AA change**	-	-	-

Key: The nucleotide position and base for the consensus sequence is listed across the top. Amino acids from the hawk consensus sequence are listed below. No amino acid replacements were observed.

## Discussion

### Differentiation and genetic diversity

Our results build upon previous phylogeographic work and show limited genetic differentiation among the *B. jamaicensis *subspecies examined. The data suggest that all three *B. jamaicensis *subspecies are quite similar genetically, and that *B. j. harlani *shows closer evolutionary affinities to *B. j. borealis *than to *B. j. calurus*. The close relationship and limited differentiation between *B. j. harlani *and *B. j. borealis *is supported by lack of significant θ_ST _and significant albeit shallow Φ_ST _estimates (0.004 and 0.059 respectively), high levels of estimated of gene flow based on both marker classes, UPGMA microsatellite groupings, Bayesian support in the microsatellite data for a single *B. j. harlani*/*borealis *population cluster, and absence of unique *B. j. harlani *mitochondrial haplotypes. These results indicate both historical and contemporary gene exchange between *B. j. borealis *and *B. j. harlani*. Conversely, θ_ST _and Φ_ST _estimates between *B. j. calurus *and the other two subspecies were significant for both marker types. Significant divergence has been observed among other North American raptor populations and subspecies. Eastern and western subspecies of *B. lineatus *(red-shouldered hawk) displayed significant divergence in microsatellite and mitochondrial data sets, and eastern and western populations of *Accipiter striatus *(sharp-shinned hawk) were found to be significantly diverged in the absence of any described subspecies [54,55]. Both studies employed the same set of microsatellite and/or mitochondrial markers as used here.

Estimates of rates of gene flow among subspecies based upon mitochondrial and microsatellite data indicate a high level of gene flow among all three *B. jamaicensis *subspecies examined. Both mitochondrial and microsatellite data sets indicate that over time a higher level of gene flow has occurred from *B. j. borealis *and *B. j. harlani *to *B. j. calurus *than from *B. j. calurus *to the other subspecies. Estimates of gene flow between *B. j. borealis *and *B. j. harlani *appear symmetrical when considering the microsatellite data; however, the mitochondrial estimates indicate greater gene flow from B. *j. borealis *to *B. j. harlani*. This pattern suggests that while rates of male migration between *B. j. borealis *and *B. j. harlani *may be equivalent, female *B. j. borealis *migration rates exceed those of *B. j. harlani*, a result consistent with a northern Midwest source for Yukon and Alaskan populations of *B. jamaicensis*.

The three recognized subspecies of *B. jamaicensis *examined here also displayed similar intra-subspecific levels of genetic diversity. Our sample of *B. j. harlani *showed equivalent levels of observed heterozygosity, allelic richness, nucleotide diversity and haplotype diversity to *B. j. calurus *and *B. j. borealis*. We did not find any unique *B. j. harlani *mitochondrial haplotypes and only identified five private microsatellite alleles; the absence of unique haplotypes and presence of few unique microsatellite alleles is also suggestive of contemporaneous gene exchange among populations representing the sampled subspecies.

Our analysis of melanistic individuals sampled in California during autumn migration indicated that they were genetically indistinguishable from *B. j. calurus*. Additionally, our analysis of sequence data from the melanocortin 1 receptor (*Mc1r*) showed no differences between melanistic and non-melanistic individuals, or between *B. j. harlani *and any *B. j. borealis *or *calurus*. This suggests that the *Mc1r *gene does not play the same role in *B. jamaicensis *melanism as in some other avian species [51,56].

### Evolutionary interpretation of genetic data

The genetic data described here do not support the historical designation of *B. j. harlani *as a distinct species [18,57]. The lack of significant differentiation between *B. j. harlani *and *B. j. borealis *in the microsatellite data suggests that gene exchange between the two subspecies occurs at a relatively high level, and the lack of reciprocal monophyly among haplotypes is inconsistent with differences expected at the species level. Furthermore, the absence of field evidence demonstrating assortative mating among *B. j. harlani *individuals relative to *B. j. calurus *or *B. j. borealis *and the apparent interbreeding with neighboring subspecies [19,58,59] also supports recognition of *B. j. harlani *as a member of *B. jamaicensis*, and not a distinct species. In fact, under some criteria, the pattern observed between *B. j. borealis *and *B. j. harlani *is inconsistent with differentiation expected among subspecies or "evolutionary significant units" [24-26]. Given the observed levels of differentiation based on traditional and Bayesian analyses of population structure and results of the MIGRATE analyses, it is possible that *B. j. harlani *represents a post-Pleistocene northern extension of *B. j. borealis *populations; however additional study is required to test this hypothesis.

The failure to detect microsatellite differentiation between *B. j. harlani *and *B. j. borealis *is not associated with lack of sensitivity in the genetic tools employed. In other studies, Bayesian analyses were able to identify fine-scale population structure between western North American populations of both *B. j. calurus *and *B. swainsoni *(Swainson's hawk) employing the same set of microsatellite markers used here [22,43]. The population divergences recovered in these examples corresponded to θ_ST _values of ~0.01 in both cases. In the case of *B. j. calurus*, within-subspecies genetic structure in western populations included fine-scale substructure between central California and the Intermountain West populations and was associated with significant morphological (overall size) differentiation. The morphological differentiation observed between these populations of a well described subspecies (*B. j. calurus*) indicates that variation in morphology alone may not accurately reflect speciation events. Given these previous findings using the same marker set, the data set used here is sensitive enough to detect species-level differentiation between *B. j. harlani *and other *B. jamaicensis *subspecies if it were present.

### Relevance of phenotype

In addition to a high incidence of melanism, *B. j. harlani *are highly variable in plumage particularly in tail pattern, extent of white below chin, spotting on back, and extent of melanism throughout body feathers [15,16,60]. The extent of variation makes description of definitive *B. j. harlani *characters difficult and no diagnostic set of *B. j. harlani *plumage characters has yet been accepted [60]. Whether the variable plumage characteristics within *B. j. harlani *also occur within other subspecies is also debated. A breeding individual observed in central California displayed many of the typical *B. j. harlani *plumage characters and many juvenile hawks observed during autumn migration in California have plumage characteristics that have been suggested as belonging to *B. j. harlani *[AC Hull, pers. comm.; WC Clark pers. comm.], yet these migrant juveniles were found to be genetically similar to the breeding populations of *B. j. calurus *from central California and the Great Basin [22], not *B. j. borealis *or *harlani*. To provide a more thorough understanding of how plumage variation in *B. jamaicensis *is correlated with nominate subspecies, additional field research is necessary to more completely categorize the range of plumage variation throughout *B. jamaicensis *and describe the geographic bounds of observed variation. Additional surveys and sampling of individuals from the Yukon, British Columbia, and prairie region would be particularly helpful in resolving the relationship between phenotype, environment, and population structure.

The mechanisms influencing plumage patterns in *B. j. harlani *require further investigation. Potential mechanisms include: 1) a *B. j. harlani*-specific gene or gene-complex, or 2) particular environmental, temporal, or physiological conditions within the *B. j. harlani *range interacting with the standard *B. jamaicensis *genotype without involvement of a unique *B. j. harlani *genotype. In light of recent research that has begun to reveal the role of environmental conditions, including temperature and oxidative stress, on melanin-based plumage [11-13,61], a controlled study of *B. j. harlani *plumage characters would be a fruitful avenue of future research.

More broadly, the underlying factors responsible for the base melanistic plumage in *B. j. harlani*, and more generally, melanism in *B. jamaicensis *require additional study. Our data suggest that the *Mc1r *locus does not play a similar role as in other avian species previously examined [51,56,62]. The gene or genes responsible for melanism in *B. jamaicensis *remain to be identified.

## Conclusions

These genetic data indicate low levels of differentiation among three recognized subspecies of *B. jamaicensis *examined, particularly between *B. j. borealis *and *B. j. harlani*. In light of previous population genetic structure investigations of species within the genus *Buteo*, our findings are inconsistent with the historical designation of *B. j. harlani *as a separate species. Rather, the mtDNA data are consistent with contemporary subspecies nomenclature and data from both marker classes suggest that gene flow is occurring at a relatively high level among these subspecies. Additional research is necessary to fully describe the geographical variation in plumage observed in *B. jamaicensis *and to determine the underlying mechanisms responsible for the variation seen in *B. j. harlani*.

## Authors' contributions

JH, ST, DM, HH, and HE designed the study. JH and ST obtained the genetic samples. JH, ST, and EK did the molecular work and obtained the sequence data. JH and ST did the phylogenetic analyses. JH, ST, and EK wrote a first draft. The final version was completed by JH, DM, ST, EK, HH, and HE. All authors read and approved the final manuscript.

## References

[B1] Sites JW Marshall JC Operational criteria for delimiting species Annu Rev Ecol Evol Syst 2004 35 199 227 10.1146/annurev.ecolsys.35.112202.130128

[B2] de Queiroz K Different species problems and their resolution BioEssays 2005 27 1263 1269 10.1002/bies.20325 16299765

[B3] Hey J On the failure of modern species concepts Trends Ecol Evol 2006 21 447 450 10.1016/j.tree.2006.05.011 16762447

[B4] Omland KE Tarr CL Boarman WI Marzluff JM Fleischer RC Cryptic genetic variation and paraphyly in ravens Proc R Soc Lond B Biol Sci 2000 267 2475 2482 10.1098/rspb.2000.1308 PMC1690844 11197122

[B5] Hull JM Savage WK Bollmer JL Kimball RT Parker PG Whiteman NK Ernest HB On the origin of the Galapagos hawk: an examination of phenotypic differentiation and mitochondrial paraphyly Biol J Linn Soc 2008 95 779 789 10.1111/j.1095-8312.2008.01082.x

[B6] Zink RM The role of subspecies in obscuring avian biological diversity and misleading conservation policy Proc R Soc Lond B Biol Sci 2004 271 561 564 10.1098/rspb.2003.2617 PMC1691635 15156912

[B7] American Ornithologists' Union Forty-second supplement to the American Ornithologists' Union *Check-list of North American Birds* Auk 2000 117 847 858 10.1642/0004-8038(2000)117[0847:FSSTTA]2.0.CO;2

[B8] Zink RM Barrowclough GF Atwood JL Blackwell-Rago RC Genetics, taxonomy, and conservation of the threatened California Gnatcatcher Conserv Biol 2000 5 1394 1405 10.1046/j.1523-1739.2000.99082.x

[B9] Johnson JA Burnham KK Burnham WA Mindell DP Genetic structure identified among continental and island populations of gyrfalcons Mol Ecol 2007 16 3145 3160 10.1111/j.1365-294X.2007.03373.x 17651193

[B10] Phillimore AB Owens IPF Are subspecies useful in evolutionary and conservation biology? Proc R Soc Lond B Biol Sci 2006 1590 1049 1053 10.1098/rspb.2005.3425 PMC1560251 16600880

[B11] Griffith SC Parker TH Olson VA Melanin versus carotenoid-based sexual signals: is the difference really so black and red? Anim Behav 2006 71 749 763 10.1016/j.anbehav.2005.07.016

[B12] McGraw KJ An update on the honesty of melanin-based color signals in birds Pigment Cell and Melanoma Res 2008 21 133 138 10.1111/j.1755-148X.2008.00454.x 18426406

[B13] Galvan I Alonso-Alvarez C The expression of melanin-based plumage is separately modulated by exogenous oxidative stress and a melanocortin Proc R Soc Lond B Biol Sci 2009 1670 3089 3097 10.1098/rspb.2009.0774 PMC2817136 19520801

[B14] Vergara P Fargallo JA Martinez-Padilla J Lemus JA Inter-annual variation and information content of melanin-based coloration in female Eurasian kestrels Biol J Linn Soc 2009 97 781 790 10.1111/j.1095-8312.2009.01263.x

[B15] Preston CR Beane RD Poole A, Gill F Red-tailed Hawk (*Buteo jamaicensis*) The Birds of North America 1993 Philadelphia: The Academy of Natural Sciences; Washington, DC: The American Ornithologists' Union 52

[B16] Clark WS Wheeler BK A Field Guide to Hawks of North America, Revised 2001 Boston: Houghton Mifflin

[B17] Clark WS Extreme variation in the tails of adult Harlan's Hawks Birding in press

[B18] Peters JL Check-list of birds of the world 1931 1 Cambridge: Harvard University Press

[B19] Ridgway R Harlan's Hawk a race of the Red-tail, and not a distinct species Auk 1890 7 205

[B20] Mindell DP Harlan's Hawk (*Buteo jamaicensis harlani*): a valid subspecies Auk 1983 100 161 169

[B21] Mindell DP Plumage variation and winter range of Harlan's Hawk (*Buteo jamaicensis harlani*) Amer Birds 1985 39 127 133

[B22] Hull JM Hull AC Sacks BN Smith JP Ernest HB Landscape characteristics influence morphological and genetic differentiation in a widespread raptor Mol Ecol 2008 17 810 824 18208488 10.1111/j.1365-294X.2007.03632.x

[B23] Pearlstine EV Variation in mitochondrial DNA of four species of migratory raptors J Raptor Res 2004 38 250 255

[B24] Ball RM Avise JC Mitochondrial-DNA phylogeographic differentiation among avian populations and the evolutionary significance of subspecies Auk 1992 109 626 636

[B25] Mortiz C Defining evolutionarily significant units for conservation Trends Ecol Evol 1994 9 373 375 10.1016/0169-5347(94)90057-4 21236896

[B26] Avise JC Walker D Pleistocene phylogeographic effects on avian populations and the speciation process Proc R Soc Lond B Biol Sci 1998 265 457 463 10.1098/rspb.1998.0317 PMC1688909 9569664

[B27] Hull JM Tufts D Topinka JR May B Ernest HB Development of 19 microsatellite loci for Swainson's hawks (*Buteo swainsoni*) and other buteos Mol Ecol Notes 2007 7 346 349 10.1111/j.1471-8286.2006.01604.x

[B28] Toonen RJ Hughes S Increased Throughput for Fragment Analysis on an ABI Prism^® ^377 Automated Sequencer Using a Membrane Comb and STRand Software Biotechniques 2001 31 1320 1324 11768661

[B29] Raymond M Rousset F GENEPOP Version 1.2: population genetics software for exact tests and ecumenicism J Hered 1995 86 248 249

[B30] Rice WR Analyzing tables of statistical tests Evolution 1989 43 223 225 10.2307/2409177 28568501

[B31] van Oosterhout C Hutchinson WF Wills DPM Shipley P MICRO-CHECKER: software for identifying and correcting genotyping errors in microsatellite data Mol Ecol Notes 2004 4 535 538 10.1111/j.1471-8286.2004.00684.x

[B32] Glaubitz JC CONVERT: a user friendly program to reformat diploid genotypic data for commonly used population genetic software packages Mol Ecol Notes 2004 4 309 310 10.1111/j.1471-8286.2004.00597.x

[B33] Park SDE Trypanotolerance in West African Cattle and the Population Genetic Effects of Selection PhD Thesis 2001 University of Dublin

[B34] Goudet J FSTAT (version 1.2): a computer program to calculate F-statistics J Hered 1995 86 485 486

[B35] Excoffier L Laval G Schneider S Arlequin ver. 3.0: An integrated software package for population genetics data analysis Evol Bioinformatics Online 2005 1 47 50 PMC2658868 19325852

[B36] Nei M Molecular Evolutionary Genetics 1987 New York: Columbia University Press

[B37] Felsenstein J PHYLIP - Phylogenetic Inference Package, Version 3.2 1989 University of Washington

[B38] Pritchard JK Stephens M Donnelly P Inference of population structure using multilocus genotype data Genetics 2000 155 945 959 10835412 10.1093/genetics/155.2.945 PMC1461096

[B39] Pritchard JK Wen W Documentation for Structure Software Version 2 http://pritch.bsd.uchicago.edu.

[B40] Waples RS Gaggiotti O What is a population? An empirical evaluation of some genetic methods for identifying the number of gene pools and their degree of connectivity Mol Ecol 2006 15 1419 1439 10.1111/j.1365-294X.2006.02890.x 16629801

[B41] Kimball RT Braun EL Zwartjes PW Crowe TM Ligon JD A molecular phylogeny of the pheasants and partridges suggests that these lineages are not monophyletic Mol Phylogenet Evol 1999 11 38 54 10.1006/mpev.1998.0562 10082609

[B42] Bollmer JL Kimball RT Whiteman NK Sarasola JH Parker PG Phylogeography of the Galapagos hawk (*Buteo galapagoensis*): A recent arrival to the Galapagos Islands Mol Phylogenet Evol 2006 39 237 247 10.1016/j.ympev.2005.11.014 16376110

[B43] Hull JM Anderson R Bradbury M Estep J Ernest HB Population structure and genetic diversity of Swainson's Hawks (*Buteo swainsoni*): implications for conservation Conservat Genet 2008 9 305 316 10.1007/s10592-007-9342-y

[B44] Swofford DL PAUP*. Phylogenetic analysis using parsimony (*and other methods). Version 4 2000 Sunderland: Sinauer

[B45] Posada D Crandall KA Modeltest: testing the model of DNA substitution Bioinformatics 1998 14 817 818 10.1093/bioinformatics/14.9.817 9918953

[B46] Akaike H A new look at the statistical model identification IEEE Transactions on Automatic Control 1974 19 716 723 10.1109/TAC.1974.1100705

[B47] Rozas J Sánchez-De JC Barrio I Messeguer X Rozas R DnaSP, DNA polymorphism analyses by the coalescent and other methods Bioinformatics 2003 19 2496 2497 10.1093/bioinformatics/btg359 14668244

[B48] Beerli P Felsenstein J Maximum likelihood estimation of migration rates and population numbers of two populations using a coalescent approach Genetics 1999 152 763 773 10353916 10.1093/genetics/152.2.763 PMC1460627

[B49] Beerli P MIGRATE: documentation and program, part of LAMARC. Version 2.0. Revised 23 December 2004 http://popgen.sc.fsu.edu/Migrate/Migrate-n.html

[B50] Beerli P Felsenstein J Maximum likelihood estimation of a migration matrix and effective population sizes in n subpopulations by using a coalescent approach Proc Natl Acad Sci USA 2001 98 4563 4568 10.1073/pnas.081068098 11287657 PMC31874

[B51] Mundy NI Badcock NS Hart T Scribner K Jansen K Nadeau NJ Conserved genetic basis of a quantitative plumage trait involved in mate choice Science 2004 303 1870 1873 10.1126/science.1093834 15031505

[B52] Hasegawa M Kishino KT Dating the human-ape splitting by a molecular clock of mitochondrial DNA J Mol Evol 1985 22 160 174 10.1007/BF02101694 3934395

[B53] Kimura M Estimation of evolutionary rate of base substitutions through comparative studies of nucleotide sequences J Mol Evol 1981 16 111 120 10.1007/BF01731581 7463489

[B54] Hull JM Strobel BN Boal CW Hull AC Dykstra CR Irish AM Fish AM Ernest HB Comparative phylogeography and population genetics within *Buteo lineatus *reveals evidence of distinct evolutionary lineages Mol Phylogenet Evol 2008 49 988 996 10.1016/j.ympev.2008.09.010 18848634

[B55] Hull JM Girman DJ Effects of Holocene climate change on the historical demography of migrating sharp-shinned hawks (*Accipiter striatus velox*) in North America Mol Ecol 2005 14 159 170 10.1111/j.1365-294X.2004.02366.x 15643959

[B56] Theron E Hawkins K Bermingham E Ricklefs RE Mundy NI The molecular basis of an avian plumage polymorphism in the wild: a melanocortin-1-receptor point mutation is perfectly associated with the melanic plumage morph of the bananaquit, *Coereba flaveola* Curr Biol 2001 11 550 557 10.1016/S0960-9822(01)00158-0 11369199

[B57] Audubon JJ Ornithological Biography 1831 Edinburgh: Adam and Charles Black

[B58] Taverner PA A study of Buteo borealis, the Red-tailed Hawk, and its varieties in Canada Victoria Memorial Museum Bulletin 1927 48

[B59] Lowe CM Certain life history aspects of the Red-tailed Hawk in central Oklahoma and interior Alaska MS thesis 1978 University of Alaska, Fairbanks

[B60] Liguori J Sullivan BL Comparison of Harlan's with western and eastern red-tailed hawks Birding 2010 42 31 37

[B61] Galeotti P Rubolini D Sacchi R Fasola M Global changes and animal phenotypic responses: melanin-based plumage redness of scops owls increased with temperature and rainfall during the last century Biol Letters 2009 5 532 534 10.1098/rsbl.2009.0207 PMC2781926 19411274

[B62] Cheviron ZA Hackett SJ Brunfield RT Sequence variation in the coding region of the melanocortin-1-receptor (MC1R) is not associated with plumage variation in the blue-crowned manakin (*Lepidothrix coronata*) Proc R Soc Lond B Biol Sci 2006 273 1613 1318 10.1098/rspb.2006.3499 PMC1704083 16769631

